# A compact PET detector module using SiPMs and MVT digitizers

**DOI:** 10.1186/2197-7364-2-S1-A7

**Published:** 2015-05-18

**Authors:** Xiang Liu, Daoming Xi, Chen Zeng, Wei Liu, Rui Chen, Xiongze Mei, Yiexuan Hua, Xiaoyao Feng, Jie Wu, Heejong Kim, Peng Xiao, Chien-Min Kao, Qingguo Xie

**Affiliations:** Huazhong University of Science & Technology, Hubei, China; University of Chicago, Chicago, Illinois USA

Nowadays the simultaneous PET/MRI is being paid more and more attentions. Since MRI provides a high resolution, unsurpassed soft tissue contrast imaging and PET reveal the metabolism progress in molecular level, it is reasonable to believe the PET/MRI will be a powerful tool to improve the understanding of pathogenesis and mechanism of brain disease. Such a simultaneous system can be implemented by integrating the RF coil with a PET scanner or inserting the PET scanner inside a MRI. In these solutions, one of the challenges is to develop compact and magnetic field compatible PET detectors. Thanks to the SiPMs, such detectors now are realizable. Aim for the simultaneous PET/MRI system, we have developed a compact PET detector module by using of the SiPMs and MVT digitizers. In the module, output signals from the LYSO/SiPM detector blocks are sampled by the MVT digitizers and then sent out via the Ethernet communicator. The timing, energy and position information will be picked up by digitally analyzing the resulted samples in a PC. In the LYSO/SiPM detector blocks, we use cross-wire method to readout the signals from SiPMs and further using transmission-line method to multiplex the signals. In this way, we get a 36:4 readout channel reducing. In this work, we have setup a dual-panel coincidence detection and imaging system by using a pair of the detector modules. The timing and energy resolution of the system are measured to be 1.67nS FWMH and 16.8%@511keV, respectively. With the system, we also obtained the imaging of a home-made mirco-Derenzo phantom which successfully resolved the 1.6 mm hollows. In future, we will investigate the feasibility of building a simultaneous PET/MRI system by using these modules.

